# A prospective 5-year study on the use of transient elastography to monitor the improvement of non-alcoholic fatty liver disease following bariatric surgery

**DOI:** 10.1038/s41598-021-83782-0

**Published:** 2021-03-08

**Authors:** Shirley Yuk-Wah Liu, Vincent Wai-Sun Wong, Simon Kin-Hung Wong, Grace Lai-Hung Wong, Carol Man-sze Lai, Candice Chuen-Hing Lam, Sally She-Ting Shu, Henry Lik-Yuen Chan, Enders Kwok-Wai Ng

**Affiliations:** 1 Department of Surgery, Prince of Wales Hospital, Faculty of Medicine, The Chinese University of Hong Kong, Hong Kong, China; 2 Institute of Digestive Disease, The Chinese University of Hong Kong, Hong Kong, China; 3 Department of Medicine and Therapeutics, Prince of Wales Hospital, Faculty of Medicine, The Chinese University of Hong Kong, Hong Kong, China

**Keywords:** Liver, Liver diseases, Obesity

## Abstract

Liver stiffness measurement (LSM) by transient elastography (TE) is a non-invasive assessment for diagnosing and staging liver fibrosis in non-alcoholic fatty liver disease (NAFLD). Evidence on its role as a longitudinal monitoring tool is lacking. This study aims to evaluate the role of TE in monitoring NAFLD improvement following bariatric surgery. This study prospectively recruited 101 morbidly obese patients undergoing laparoscopic bariatric surgery for intraoperative liver biopsy. Thirty-seven patients of the cohort received perioperative TE. Postoperative anthropometric, biochemical and LSM data were collected annually for 5 years. In 101 patients receiving liver biopsy (mean age 40.0 ± 10.3 years, mean body-mass-index (BMI) 40.0 ± 5.7 kg/m^2^), NASH and liver fibrosis were diagnosed in 42 (41.6%) and 48 (47.5%) patients respectively. There were 29 (28.7%) stage 1, 11 (10.9%) stage 2, 7 (6.9%) stage 3, and 1 (1.0%) stage 4 fibrosis. In 37 patients receiving TE (mean age 38.9 ± 10.8 years, mean BMI 41.1 ± 5.6 kg/m^2^), the percentages of total weight loss were 21.1 ± 7.6% at 1 year, 19.7 ± 8.3% at 3 years, and 17.1 ± 7.0% at 5 years after surgery. The mean LSM reduced significantly from 9.8 ± 4.6 kPa at baseline to 6.9 ± 3.4 kPa at 1 year, 7.3 ± 3.0 kPa at 3 years, and 6.8 ± 2.6 kPa at 5 years (*P* = 0.002). Using pre-defined LSM cut-offs, the rates of significant fibrosis, advanced fibrosis and cirrhosis being ruled out at 5 years improved from baseline values of 43.7 to 87.5% (*P* < 0.001), 56.8 to 91.7% (*P* < 0.001), and 64.9 to 91.7% (*P* < 0.001), respectively. TE was a useful monitoring tool in demonstrating the improvement of liver fibrosis following bariatric surgery.

## Introduction

Non-alcoholic fatty liver disease (NAFLD) is a spectrum of chronic liver diseases characterized by liver steatosis, steatohepatitis (NASH) and liver fibrosis due to over-nutrition and its associated metabolic syndrome^1^. With the rising global epidemic of obesity, the prevalence of NAFLD has increased rapidly over the last two decades^2^. Bariatric surgery is currently recommended as a standard treatment for obese type 2 diabetes mellitus (T2DM)^3^. Because NAFLD represents the hepatic manifestation of metabolic syndrome, bariatric surgery is increasingly advocated as a treatment for NAFLD^4–6^. Despite the lack of prospective randomized trials comparing the effects of bariatric surgery with other interventions on NAFLD, recent systematic reviews and meta-analyses have demonstrated the benefits of bariatric surgery in normalizing liver biochemistry and reversing histological markers of NAFLD^7–10^.

To evaluate the efficacy of bariatric surgery in treating NAFLD, an optimal assessment tool has not yet been established. While most studies rely on liver biochemistry as surrogate markers of NAFLD, normal liver enzyme levels cannot exclude the disease^7–10^. To date, histological assessment by liver biopsy is regarded as the gold standard in diagnosing different degrees of NAFLD and confirming the improvement of NAFLD in response to interventions^1,4–6,11,12^. Being an invasive assessment with concerns on complications, high execution cost, sampling error and inter-observer variability, repeated liver biopsies cannot be recommended to every patient after bariatric surgery^13^. Hence, other non-invasive modalities, such as imaging techniques, are warranted for follow-up monitoring of NAFLD. Ultrasonography and magnetic resonance imaging (MRI) are commonly employed for the initial diagnosis and subsequent monitoring of NAFLD in an attempt to reduce the need of liver biopsy. Although ultrasonography offers the advantages of wide availability and low cost, its sensitivity in detecting liver steatosis in morbidly obese patients is disappointing^14^. While MRI is a sensitive tool in quantifying liver steatosis even in morbidly obese patients and magnetic resonance elastography (MRE) is highly accurate in detecting liver fibrosis, both techniques are largely limited by high costs and low accessibility^14^.

Vibration-controlled transient elastography (TE) is one of the available non-invasive assessment tools for NAFLD. It works by generating vibrations of mild amplitude and low frequency to induce elastic shear waves that propagate through liver tissues for stiffness measurement^15^. Newer models currently allow simultaneous quantification of liver fibrosis by liver stiffness measurement (LSM) and liver steatosis by controlled attenuation parameter (CAP)^16^. TE provides multiple advantages of low cost, short procedure time, immediate result availability, good reproducibility and the ability to be performed in an outpatient setting. Its roles in diagnosing NAFLD and assessing the severity of NAFLD have been extensively investigated in cross-sectional studies^15,17–20^. However, evidences on its role as a monitoring tool in longitudinal studies are largely lacking^21–25^. Whether TE can serve as a follow-up assessment tool after bariatric surgery or other interventions remains to be elucidated. This study aims to evaluate the role of TE as a monitoring tool for the improvement of NAFLD in patients undergoing bariatric surgery.

## Material and methods

### Patients

This was a prospective longitudinal observational study on consecutive adults who received laparoscopic adjustable gastric banding (LAGB), laparoscopic sleeve gastrectomy (LSG), and laparoscopic Roux-en-Y gastric bypass (RYGB) for the treatment of morbid obesity between January 2011 and February 2013. Following the International Federation for the Surgery of Obesity and Metabolic Disorders—Asian Pacific Chapter consensus statement 2011, patients with a body-mass-index (BMI) ≥ 35 kg/m^2^ regardless of the existence of comorbidities or BMI ≥ 30 kg/m^2^ with inadequately controlled T2DM or metabolic syndrome were eligible for surgery^26^. We excluded patients who had (i) excessive alcohol consumption > 140 g/week in men and > 70 g/week in women; (ii) positive hepatitis B surface antigen or anti-hepatitis C virus antibody; (iii) secondary causes of liver steatosis including autoimmune or metabolic causes; (iv) been using drugs that could induce liver steatosis or insulin sensitization, such as corticosteroids, methotrexate, and tamoxifen; (iv) prior hepatocellular carcinoma or liver resection; (v) any malignancy in the preceding 5 years, and (vi) the operation performed as a revisional procedure from other types of bariatric surgery. The study protocol was approved by the Joint Chinese University of Hong Kong—New Territories East Cluster Clinical Research Ethics Committee and was conducted in accordance with the ethical standards of the Helsinki Declaration of 1964 and its later versions. Written informed consent was obtained from all patients for study enrolment.

### Operative technique

The decision on the type of surgery was made by the patients after a detailed counselling on the risks and benefits of different procedures by the bariatric surgeons. All procedures were performed by two experienced bariatric surgeons. Our techniques of LAGB, LSG and RYGB were previously described^27–29^.

### Laparoscopic liver biopsy

Transperitoneal liver biopsy was performed on all patients during laparoscopic bariatric surgery. A liver sample of ≥ 15 mm long was taken from left hepatic lobe using 16-gauge Temno™ II semi-automatic biopsy needle (Cardinal Health, Dublin, Ohio, United States). It was evaluated by one experienced histopathologist after preparation with hematoxylin & eosin stain and Masson’s trichrome stain. Histopathological findings were reported using Non-Alcoholic Steatohepatitis Clinical Research Network Scoring System^30^. The NAFLD activity score (NAS) was the sum of scores for hepatic steatosis (score S0–3), lobular inflammation (score L0–3) and hepatocyte ballooning (score B0–2). In this study, NASH was defined using the histopathological algorithm for morbidly obese patients proposed by Bedossa et al.^31^ NASH was defined as present when the three scores for steatosis, lobular inflammation and hepatocellular ballooning were all ≥ 1. Fibrosis was staged from 0 to 4: stage F0, no fibrosis; F1, peri-sinusoidal or peri-portal fibrosis; F2, peri-sinusoidal and portal/peri-portal fibrosis; F3, bridging fibrosis; and F4, cirrhosis^5^. Stage F2 or above was defined as significant fibrosis (SF) and stage F3 or above was defined as advanced fibrosis (AF).

### Transient elastography

Our study protocol was revised in the latter third of the study to add perioperative TE for LSM in the recruited patients. TE by FIBROSCAN (Echosens, Paris, France) was performed within 4 weeks prior to bariatric surgery and then annually for 5 years after surgery. All examinations were performed by a single officially-trained operator who had independently performed over 50 examinations. After prior fasting for 6 h, LSM was conducted using M and XL probes being placed between intercostal spaces over right hepatic lobe when patients were lying in dorsal decubitus position with their right arms in maximal abduction. For every patient, ten valid acquisitions were obtained. The median value was used to represent the liver elastic modulus in kilopascal (kPa). The interquartile range (IQR) of LSM was taken as the interval containing 50% of valid measurements between 25 and 75th percentiles. The success rate was calculated by dividing the number of valid acquisitions with the total number of acquisitions. LSM was considered reliable if ≥ 10 valid acquisitions were obtained and the IQR-to-median ratio of LSM was ≤ 0.3. At baseline, both M and XL probes were used. During subsequent follow-up assessments, M probe was used for patients with BMI < 30 kg/m^2^ and XL probe was used for those having BMI ≥ 30 kg/m^2^. If reliable results could not be obtained from M probe, additional examination using XL probe was conducted. Because one patient might have reliable TE obtained by both M and XL probes, an overall LSM was chosen from reliable results by XL probe in patients having BMI ≥ 30 kg/m^2^ or from reliable results by M probe in those having BMI < 30 kg/m^2^.

### Follow-up assessment

All patients recruited for TE were prospectively assessed at baseline and then followed annually for 5 years after surgery. During each assessment, anthropometric measurement of body weight, BMI, percentage of body fat, and waist circumference was conducted. Body fat percentage was estimated by an impedance body composition analyser (X-Scan Plus, Accuniq, Seoul, Korea). The percentage of total weight loss (%TWL) and the percentage of excess weight loss (%EWL) were used to represent the extent of weight reduction. %EWL was calculated using a BMI value of 25 kg/m^2^ as the ideal body weight. Biochemical parameters including total bilirubin, alkaline phosphatase (ALP), alanine transaminase (ALT), gamma-glutamyl transferase (GGT), fasting blood glucose (FBG), glycosylated hemoglobin (A1c), total cholesterol (TC), triglyceride (TG), high density lipoprotein cholesterol (HDL-C), low density lipoprotein cholesterol (LDL-C), C reactive protein (CRP), creatinine, albumin, platelet count and mean platelet volume were measured after overnight fasting.

### Primary outcome

The primary outcome was the changes in the proportions of patients having significant fibrosis ruled out, advanced fibrosis ruled out, and cirrhosis ruled out before and after bariatric surgery as measured by LSM cut-off. Because different LSM cut-off values were being reported for NAFLD with different sensitivities and specificities in different population cohorts, this study adopted the cut-off values as previously reported by our group^32,33^. These same cut-off values were adopted and recommended by the World Federation for Ultrasound in Medicine and Biology Guidelines^34^. For M probe, the cut-off values used to rule out significant fibrosis, advanced fibrosis and cirrhosis were < 9.0 kPa, < 9.6 kPa, and < 11.5 kPa, respectively^32^. For XL probe, the cut-off values used to rule out significant fibrosis, advanced fibrosis and cirrhosis were < 8.2 kPa, < 9.3 kPa, and < 11.0 kPa, respectively^33^.

### Secondary outcomes

Several other measures were assessed as secondary outcomes. The baseline prevalence of NASH and liver fibrosis in the whole cohort receiving intraoperative liver biopsy was estimated. In patients receiving TE, the postoperative changes in weight parameters, LSM, and liver biochemistry were assessed. The rates of patients having LSM improvement during follow-up were also estimated. LSM improvement was defined as having ≥ 30% reduction from baseline in the overall LSM measured. Comparison of the changes in the clinical and biochemical parameters between patients with and without LSM improvement at 1 year and 5 years after bariatric surgery was also conducted.

### Statistical analysis

Statistical analysis was performed using IBM SPSS STATISTICS version 25 (IBM, New York, United States). Changes in postoperative weight loss outcomes and biochemical parameters were compared by Wilcoxon signed rank test and Friedman test. Chi-square test or Fisher exact test was used for comparison of nominal and categorical variables. The area under the curve (AUC) of a receiver operating characteristics (ROC) curve was used to assess the performance of LSM as a diagnostic test for liver fibrosis. A two-sided *P* value of < 0.05 was regarded as significant. Follow-up data of those with lost-to-follow-up were not included in the analyses.

## Results

### Baseline characteristics

In this study, 101 ethnic Chinese morbidly obese patients (38 males and 63 females) received intraoperative liver biopsy during laparoscopic bariatric surgery (39 LAGB, 57 LSG and 5 RYGB). A total of 37 patients (17 males and 20 females) consented for LSM and received TE before and after surgery (9 LAGB, 25 LSG and 3 RYGB). The baseline characteristics and biochemical variables of the cohort with LSM (n = 37) are summarized in Table 1.

**Table 1:** Baseline characteristics of the patient cohorts.

	Patients receiving intraoperative liver biopsy and perioperative LSM (N = 37)
Age (years)	38.9 ± 10.8
Gender (male : female)^a^	17 (45.9) : 20 (54.1)
Body weight (kg)	108.8 ± 17.9
Body mass index (kg/m^2^)	41.1 ± 5.6
Body fat percentage (%)	48.5 ± 11.3
Waist circumference (cm)	123.5 ± 13.5
Metabolic syndrome^a^	16 (43.2)
Type 2 diabetes mellitus^a^	18 (48.6)
Pre-diabetes^a^	7 (18.9)
Hypertension^a^	19 (51.4)
Dyslipidemia^a^	16 (43.2)
Osteoarthritis^a^	20 (54.1)
Obstructive sleep apnea^a^	21 (56.8)
Gastroesophageal reflux disease^a^	6 (16.2)
Polycystic ovarian syndrome^a^	6/20 (30.0)
Bilirubin (umol/L)	11.5 ± 4.1
Alkaline phosphatase (IU/L)	69.9 ± 16.6
Alanine transferase (IU/L)	45.9 ± 25.4
Gamma glutamyl transferase (U/L)	54.3 ± 37.2
Fasting blood glucose (mmol/L)	6.9 ± 2.9
Glycosylated hemoglobin (%)	7.3 ± 1.8
Total cholesterol (mmol/L)	4.9 ± 1.1
Triglyceride (mmol/L)	1.7 ± 0.7
High density lipoprotein cholesterol (mmol/L)	1.2 ± 0.3
Low density lipoprotein cholesterol (mmol/L)	3.0 ± 1.0
C reactive protein (ug/ml)^b^	3.9 (2.5–7.0)
Creatinine (umol/L)	66.8 ± 16.9
Albumin (g/L)	43.5 ± 2.7
Platelet count (× 10^9^/L)	270.4 ± 58.6
Mean platelet volume (fL)	8.7 ± 0.8
***Liver biopsy findings***^a^	
**Steatosis**^c^	
S0	1 (2.7%)
S1	11 (29.7%)
S2	14 (37.8%)
S3	11 (29.7%)
**Lobular inflammation**^c^	
L0	6 (16.2%)
L1	27 (73.0%)
L2	4 (10.8%)
L3	0 (0%)
**Hepatocellular ballooning**^c^	
B0	14 (37.8%)
B1	21 (56.8%)
B2	2 (5.4%)
**NAFLD activity score**^c^	
0	1 (2.7%)
1	3 (8.1%)
2	5 (13.5%)
3	8 (21.6%)
4	8 (21.6%)
5	9 (24.3%)
6	3 (8.1%)
7	0 (0%)
8	0 (0%)
**Fibrosis stage**^d^	
F0	13 (35.1%)
F1	14 (37.8%)
F2	6 (16.2%)
F3	3 (8.1%)
F4	1 (2.7%)
**NASH**^e^	23 (62.2%)

Data are mean ± standard deviations unless otherwise specified. LSM, liver stiffness measurement; NASH, non-alcoholic steatohepatitis.

^a^Count (percentage).

^b^Median (interquartile range).

^c^Hepatic steatosis was graded by the percentage of parenchymal involvement with steatosis under low- to medium-power evaluation: score S0, steatosis < 5%; score S1, steatosis 5–33%; score S2, steatosis 33–66%; and score S3, steatosis > 66%. Lobular inflammation was assessed by the number of inflammatory foci per 200-power field: score L0, no foci; score L1, < 2 foci; score L2, 2–4 foci; and score L3, > 4 foci. Hepatocellular ballooning was graded as B0 for no ballooning, B1 for few balloon cells, and B2 for many cells or prominent ballooning. NAFLD activity score was the sum of scores for hepatic steatosis (score S0–3), lobular inflammation (score L0–3) and hepatocyte ballooning (score B0–2).

^d^NASH was defined as steatosis score ≥ 1 plus lobular inflammation score ≥ 1 plus hepatocellular ballooning score ≥ 1.

^e^Fibrosis was staged from 0 to 4: stage F0, no fibrosis; F1, peri-sinusoidal or peri-portal fibrosis; F2, peri-sinusoidal and portal/peri-portal fibrosis; F3, bridging fibrosis; and F4, cirrhosis.

### Baseline prevalence of NAFLD in whole cohort

Liver biopsy was successfully performed without perioperative complications in all 101 patients. Forty-two (41.6%) patients were diagnosed to have NASH. A total of 62 (61.4%) patients and 24 (23.8%) patients had NAS ≥ 3 and NAS ≥ 5 respectively. Forty-eight (47.5%) patients had different degrees of liver fibrosis. The prevalence of SF, AF and cirrhosis was 18.8% (n = 19), 7.9% (n = 8) and 1.0% (n = 1) respectively.

### Baseline performance of pre-defined LSM cut-offs

At baseline, all 37 patients successfully completed LSM by TE. The degree of liver fibrosis upon liver biopsy was significantly correlated with the baseline LSM results (r = 0.415, *P* = 0.015). Using pre-defined LSM cut-off values, 16 (43.2%) patients had SF ruled out, 21 (56.8%) patients had AF ruled out, and 24 (64.9%) patients had cirrhosis ruled out. Using liver biopsy, SF was absent in 27 (73.0%) patients, AF was absent in 33 (89.2%) patients, and cirrhosis was absent in 36 (97.3%) patients. For the diagnostic sensitivity of the pre-defined cut-off values, it was 51.9% for ruling out SF, 57.6% for ruling out AF, and 66.7% for ruling out cirrhosis. As for the diagnostic specificity, it was 80.0% for ruling out SF, 75.0% for ruling out AF, and 100% for ruling out cirrhosis. LSM was a fair diagnostic tool for ruling out SF (AUC = 0.715, 95%C.O. 0.517–0.913, *P* = 0.047) but not for ruling out AF (AUC = 0.663, 95%C.I. 0.390–0.936, *P* = 0.293).

### Postoperative rates of different fibrosis degrees using pre-defined LSM cut-offs

**Figure 1:**
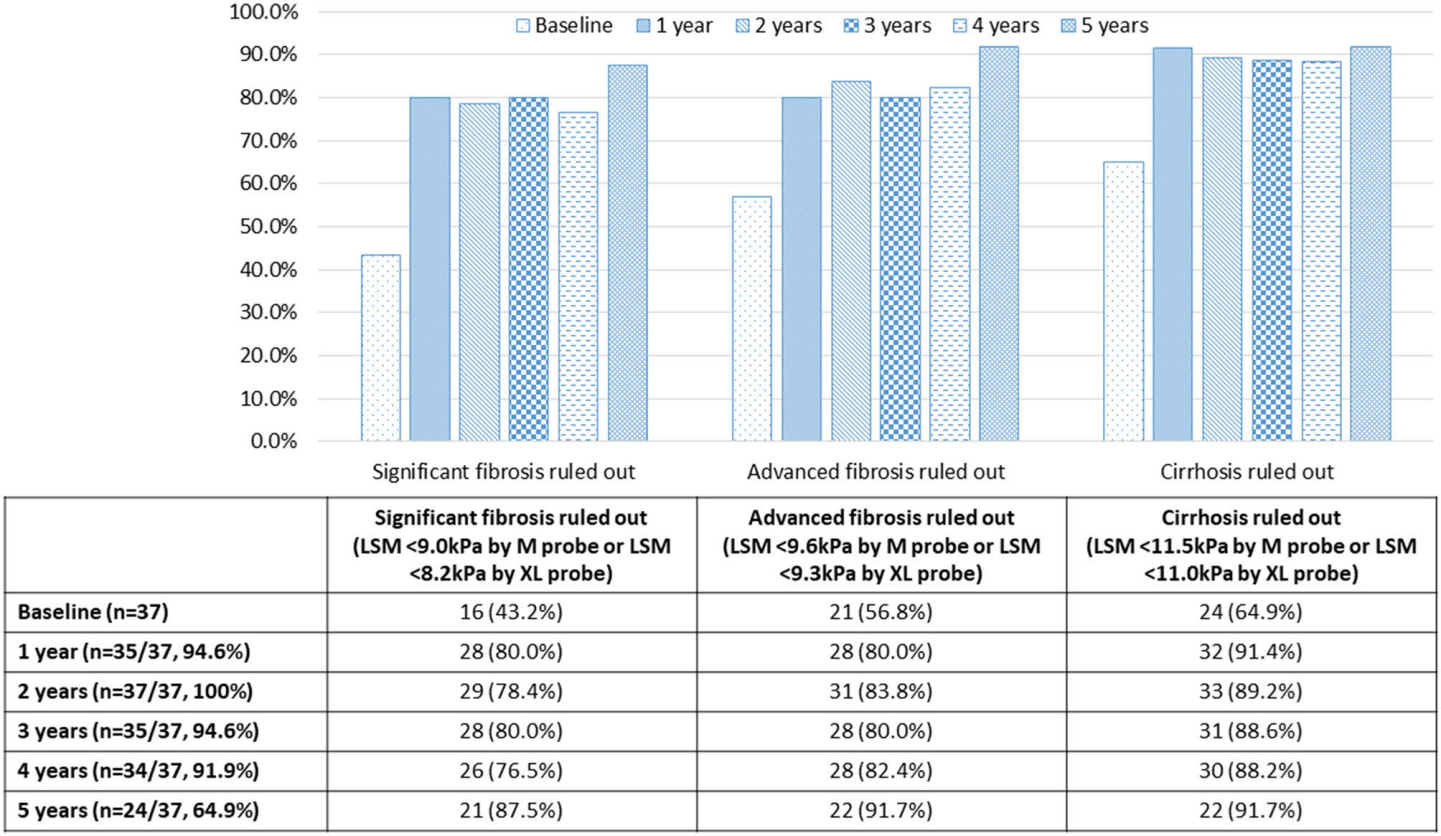
Postoperative rates of significant fibrosis ruled out, advanced fibrosis ruled out and cirrhosis ruled out using the pre-defined LSM cut-off.

Among 37 patients receiving TE, the rates of follow-up after surgery were 94.6% (n = 35/37) at 1 year, 100% (n = 37/37) at 2 years, 94.6% (n = 35/37) at 3 years, 91.9% (n = 34/37) at 4 years, and 64.9% (n = 24/37) at 5 years. The postoperative rates of ruling out SF, AF and cirrhosis by the overall LSM results are presented in Fig. 1. The rates of patients having SF ruled out by LSM improved from 43.2% at baseline to 80.0% at 1 year (*P* = 0.001), 80.0% at 3 years (*P* = 0.001), and 87.5% at 5 years (*P* < 0.001). Similarly, the rates of patients having AF ruled out by LSM increased from 56.8% at baseline to 80.0% at 1 year (*P* = 0.001), 80.0% at 3 years (*P* = 0.001), and 91.7% at 5 years (*P* < 0.001). The rates of patients having cirrhosis ruled out by LSM also improved significantly from 64.9% at baseline to 91.4% at 1 year (*P* < 0.001), 88.6% at 3 years (*P* < 0.001), and 91.7% at 5 years (*P* < 0.001).

### Postoperative LSM improvement

There was significant reduction in the overall LSM results from 9.8 ± 4.6 kPa at baseline to 6.9 ± 3.4 kPa at 1 year, 7.3 ± 3.0 kPa at 3 years, and 6.8 ± 2.6 kPa at 5 years (*P* = 0.002) (Table 2). The rates of patients having LSM improvement (≥ 30% reduction in LSM level) were 54.3% (19/35) at 1 year, 48.6% (17/35) at 3 years, and 45.8% (11/24) at 5 years. After surgery, the rates of reliable LSM results obtained by M probe increased significantly from 32.4% at baseline to 84.0% at 1 year, 96.6% at 3 years, and 100% at 5 years (*P* < 0.001). While there was a decrease in the number of patients being eligible to use XL probe after surgery due to BMI reduction, the rates of reliable results achieved were similar throughout the five postoperative years.

**Table 2:** LSM results of transient elastography by XL probe and M probe after bariatric surgery.

	Baseline (n = 37)	1 year (n = 35/37, 94.6%)	2 years (n = 37/37, 100%)	3 years (n = 35/37, 94.6%)	4 years (n = 34/37, 91.9%)	5 years (n = 24/37, 64.9%)
**XL probe**						
Patients receiving test	37	22	14	8	9	6
Patients with reliable test	35 (94.6%)	20 (90.9%)	12 (85.7%)	8 (100%)	9 (100%)	6 (100%)
LSM (kPa)^a^	9.7 ± 4.4	7.3 ± 4.0	8.8 ± 6.2	7.7 ± 4.4	8.0 ± 3.0	7.2 ± 4.0
**M probe**						
Patients receiving test	37	25	28	29	25	18
Patients with reliable test	12 (32.4%)	21 (84.0%)	24 (85.7%)	28 (96.6%)	25 (100%)	18 (100%)
LSM (kPa)^a^	10.7 ± 5.4	6.5 ± 2.0	6.8 ± 2.7	7.0 ± 2.5	6.7 ± 2.9	6.7 ± 2.2
**Overall**^b^						
Patients receiving test	37	35	37	35	34	24
Patients with reliable results	37 (100%)	35 (100%)	35 (94.6%)	35 (100%)	34 (100%)	24 (100%)
LSM (kPa)^a^	9.8 ± 4.6	6.9 ± 3.4	7.4 ± 4.3	7.3 ± 3.0	7.0 ± 3.0	6.8 ± 2.6
LSM improvement (≥ 30% reduction from baseline)	–	19 (54.3%)	17 (48.6%)	17 (48.5%)	12 (35.3%)	11 (45.8%)

Data are counts (percent) unless otherwise specified. LSM, liver stiffness measurement.

^a^LSM results (mean ± standard deviation) from reliable tests.

^b^Overall results from tests using XL probe for patients with BMI ≥ 30 kg/m^2^ or from tests using M probe for patients with BMI < 30 kg/m^2^.

### Postoperative weight and biochemical improvement

As demonstrated in Table 3, there was significant reduction in body weight, BMI, percentage of body fat and waist circumference after surgery (all *P* < 0.001). The %EWL was 58.6 ± 22.8% at 1 year, 53.9 ± 23.1% at 3 years, and 47.1 ± 21.4% at 5 years. The %TWL was 21.1 ± 7.6% at 1 year, 19.7 ± 8.3% at 3 years, and 17.1 ± 7.0% at 5 years. Both %EWL and %TWL were maintained without significant reduction till 5 years (both *P* = 0.149). Regarding liver biochemistry, there was significant reduction in ALT (*P* < 0.001) and GGT (*P* < 0.001) while serum bilirubin and ALP remained unchanged. For metabolic parameters, significant improvement was observed in FBG (*P* = 0.002), A1c (*P* < 0.001), TG (*P* < 0.001), HDL-C (*P* = 0.005), and CRP (*P* < 0.001) levels. Although serum creatinine levels showed statistically significant change (*P* = 0.002), all the postoperative creatinine results were within normal range. There was no change in the postoperative results of serum albumin, platelet count and mean platelet volume.

**Table 3:** Changes in anthropometric and biochemical outcomes after bariatric surgery.

	Baseline (n = 37)	1 year (n = 35/37, 94.6%)	2 years (n = 37/37, 100%)	3 years (n = 35/37, 94.6%)	4 years (n = 34/37, 91.9%)	5 years (n = 24/37, 64.9%)	*P* value^b^
Body weight (kg)	108.8 ± 17.9	84.2 ± 15.0	86.6 ± 19.0	86.1 ± 17.1	87.7 ± 18.1	90.3 ± 18.0	< 0.001
Body mass index (kg/m^2^)	41.1 ± 5.6	31.9 ± 4.7	32.7 ± 6.1	32.7 ± 5.4	33.3 ± 5.7	33.8 ± 5.3	< 0.001
Percentage of excess weight loss (%)	0	58.6 ± 22.8	56.1 ± 27.1	53.9 ± 23.1	51.4 ± 22.6	47.1 ± 21.4	–
Percentage of total weight loss (%)	0	21.1 ± 7.6	20.5 ± 9.5	19.7 ± 8.3	19.1 ± 8.3	17.1 ± 7.0	–
Body fat percentage (%)	48.5 ± 11.3	35.9 ± 9.7	35.8 ± 10.9	33.5 ± 6.8	32.2 ± 7.6	32.7 ± 7.8	< 0.001
Waist circumference (cm)	123.5 ± 13.5	105.3 ± 13.4	104.4 ± 15.9	103.6 ± 14.1	105.6 ± 14.7	107.8 ± 14.2	< 0.001
Bilirubin (umol/L)	11.5 ± 4.1	13.7 ± 9.6	13.3 ± 7.0	12.6 ± 5.6	12.5 ± 6.9	11.5 ± 4.4	0.869
Alkaline phosphatase (IU/L)	69.9 ± 16.6	68.6 ± 18.0	64.8 ± 14.0	65.7 ± 16.4	67.8 ± 15.5	69.2 ± 19.6	0.339
Alanine transferase (IU/L)	45.9 ± 25.4	22.4 ± 10.7	22.3 ± 14.1	23.4 ± 13.0	23.4 ± 12.0	24.5 ± 11.8	< 0.001
Gamma glutamyl transferase (U/L)	54.3 ± 37.2	30.8 ± 33.4	25.9 ± 15.7	27.3 ± 15.5	27.6 ± 17.9	26.7 ± 14.5	< 0.001
Fasting blood glucose (mmol/L)	6.9 ± 2.9	5.2 ± 1.2	5.4 ± 1.6	6.2 ± 2.6	5.8 ± 2.1	5.7 ± 1.1	0.002
Glycosylated hemoglobin (%)	7.3 ± 1.8	5.9 ± 0.7	6.1 ± 1.1	6.2 ± 1.2	6.4 ± 1.4	6.4 ± 1.3	< 0.001
Total cholesterol (mmol/L)	4.9 ± 1.1	4.8 ± 1.0	4.7 ± 0.9	4.5 ± 1.2	4.6 ± 1.1	5.0 ± 1.1	0.748
Triglyceride (mmol/L)	1.7 ± 0.7	1.1 ± 0.5	1.2 ± 0.6	1.4 ± 0.9	1.4 ± 1.0	1.4 ± 0.8	< 0.001
High density lipoprotein cholesterol (mmol/L)	1.2 ± 0.3	1.6 ± 0.5	1.5 ± 0.4	1.5 ± 0.5	1.5 ± 0.5	1.6 ± 0.5	0.005
Low density lipoprotein cholesterol (mmol/L)	3.0 ± 1.0	2.7 ± 0.7	2.7 ± 0.8	2.6 ± 0.8	2.7 ± 0.8	2.9 ± 0.8	0.778
C reactive protein (ug/ml)^a^	3.9 (2.5–7.0)	0.9 (0.6–2.1)	0.9 (0.6–2.2)	1.4 (0.6–2.7)	1.3 (0.6–2.7)	1.9 (0.8–2.7)	< 0.001
Creatinine (umol/L)	66.8 ± 16.9	66.8 ± 17.6	65.9 ± 16.6	69.8 ± 20.5	72.3 ± 21.6	71.1 ± 24.2	0.002
Albumin (g/L)	43.5 ± 2.7	43.7 ± 2.7	43.2 ± 2.6	43.0 ± 2.6	42.7 ± 2.9	41.4 ± 4.4	0.350
Platelet count (× 10^9^/L)	270.4 ± 58.5	253.0 ± 58.6	251.0 ± 58.3	254.6 ± 63.0	249.1 ± 59.4	251.2 ± 51.4	0.056
Mean platelet volume (fL)	8.7 ± 0.8	8.6 ± 0.8	8.6 ± 0.7	8.6 ± 0.7	8.6 ± 0.8	8.6 ± 0.7	0.221

Data are mean ± standard deviations unless otherwise specified.

^a^Median (interquartile range).

^b^Statistical analysis by Friedman test.

### Comparison between patients with and without LSM improvement

As shown in Table 4, postoperative LSM improvement at 1 year was significantly associated with higher percentages of FBG improvement (median 26.9% vs 8.7%, *P* = 0.020) and A1c improvement (median 19.4% vs 7.7%, *P* = 0.020). There was no association with weight loss, body fat reduction, changes in the biochemical variables or other components of metabolic syndrome. At 1 year, LSM improvement was detected in 70.6% (N = 12/17) of T2DM patients, 50.0% (N = 3/6) of pre-diabetic patients, and 33.3% (N = 4/12) of non-diabetic patients (*P* = 0.132). At 5 years, there was no significant association between LSM improvement and all measured clinical and biochemical variables.

**Table 4:** Postoperative changes in clinical and biochemical factors associated with LSM improvement at 1 year and 5 years.

	1 year			5 years		
	No LSM improvement N = 16 (45.7%)	LSM improvement N = 19 (54.3%)	*P* value	No LSM improvement N = 13 (54.2%)	LSM improvement N = 11 (45.8%)	*P* value
Percentage of excess weight loss (%)	60.9 ± 27.6	56.7 ± 18.4	0.567	50.7 + 24.5	42.9 = 17.1	0.459
Percentage of total weight loss (%)	20.6 ± 8.0	21.6 ± 7.4	0.756	16.7 = 7.2	17.5 = 7.0	0.820
Body fat percent reduction (%)	22.6 ± 17.7	26.2 ± 13.5	0.528	− 47.8 (− 78.9 to − 25.0)	− 44.7 (− 75.9 to − 38.8)	1.000
Waist circumference reduction (%)	13.8 ± 10.2	13.7 ± 8.0	0.935	13.2 = 7.0	12.8 = 8.3	0.740
Total cholesterol decreased (%)^a^	4.8 (− 9.9 to 16.3)	0 (− 22.5to 20.0)	0.502	0 (− 9.3 to 23.2)	− 7.6 (− 19.2 to 0.1)	0.088
Triglyceride decrease (%)^a^	28.3 (15.1 to 40.0)	27.3 (6.3 to 52.4)	0.987	16.7 (− 16.5 to 41.7)	8.3 (− 18.8 to 25.0)	0.494
High density lipoprotein cholesterol increase (%)^a^	29.2 (1.4 to 54.2)	15.4 (7.7 to 42.9)	0.987	30.8 (0 to 43.9)	27.3 (− 10.0 to 57.1)	0.910
Low density lipoprotein cholesterol decrease (%)^a^	12.4 (− 3.3 to 26.5)	5.9 (− 35.7 to 19.4)	0.350	7.1 (− 7.2 to 24.8)	− 5.9 (− 46.7 to 4.7)	0.072
Fasting blood glucose decrease (%)^a^	8.7 (− 4.2 to 16.1)	26.9 (2.1 to 38.7)	0.020	2.1 (− 4.3 to 23.4)	25.8 (3.9 to 37.5)	0.148
Glycosylated hemoglobin decrease (%)^a^	7.7 (1.7 to 14.6)	19.4 (9.5 to 32.4)	0.020	10.8 (4.4 to 18.1)	12.2 (7.9 to 20.5)	0.531
Systolic blood pressure decrease (%)^a^	5.1 (− 1.2 to 13.4)	10.9 (− 1.8 to 14.6)	0.441	4.5 (3.0 to 12.0)	14.2 (2.6 to 21.5)	0.197
Diastolic blood pressure decrease (%)^a^	8.0 (0.8 to 13.9)	5.3 (− 9.4 to 27.2)	0.612	− 2.3 (− 10.8 to 4.1)	1.7 (− 5.9 to 10.3)	0.223
Bilirubin decrease (%)^a^	− 12.5 (− 45.0 to 11.5)	− 14.3 (− 28.6 to 20.0)	0.589	− 8.3 (− 49.2 to 11.3)	− 16.7 (− 30.0 to 18.2)	0.733
Alkaline phosphatase decrease (%)^a^	− 2.4 (− 10.5 to 7.3)	7.4 (1.3 to 13.8)	0.067	4.9 (− 8.9 to 14.2)	3.3 (− 17.1 to 12.5)	0.776
Alanine transferase decrease (%)^a^	40.3 (7.6 to 63.2)	53.1 (31.0 to 71.2)	0.422	13.0 (4.4 to 47.9)	52.1 (16.4 to 69.0)	0.096
Gamma glutamyl transferase decrease (%)^a^	45.2 (31.4 to 57.3)	51.1 (33.6 to 67.3)	0.502	38.1 (24.0 to 54.8)	48.0 (25.0 to 65.5)	0.459
C reactive protein decrease (%)^a^	32.2 (16.7 to 79.3)	73.1 (47.4 to 85.7)	0.117	36.9 (25.2 to 67.1)	37.5 (− 10.3 to 68.1)	0.651
Creatinine decrease (%)^a^	0.6 (− 12.9 to 5.8)	4.5 (− 14.9 to 11.9)	0.502	2.3 (− 8.1 to 12.1)	12.8 (1.7 to 21.7)	0.167
Albumin increase (%)^a^	0 (− 2.3 to 4.1)	0 (− 4.5 to 7.1)	0.683	0 (− 7.8 to 5.9)	4.3 (− 9.8 to 10.5)	0.691
Platelet count decrease (%)^a^	5.7 (4.6 to 20.2)	6.3 (2.5 to 10.8)	0.523	11.0 (0.3 to 15.7)	3.2 (7.3 to 13.2)	0.277
Mean platelet volume decrease (%)^a^	3.2 (− 7.8 to 7.1)	2.1 (1.1 to 3.8)	0.949	0.5 (− 8.0 to 4.3)	1.7 (− 8.4 to 7.0)	0.683

Data are percentages of improvement of the parameters at 1 year and 5 years presented as mean ± standard deviation.

^a^Percentages of change of the parameters in median (interquartile range).

LSM, liver stiffness measurement.

## Discussion

To evaluate the effectiveness of bariatric surgery in treating NAFLD, measuring the improvement in liver fibrosis is crucial for predicting prognosis^35^. Liver biopsy is recommended as the gold standard in determining treatment responses or disease progression during NAFLD management^30^. However, when liver biopsy is used to monitor responses to bariatric surgery, the long-term compliance rate is often disappointing. In two longitudinal studies investigating the impact of bariatric surgery on NAFLD improvement, the compliance rates to repeat liver biopsy at 1 to 5 years were 47.2 to 68.9% only^36,37^. Because of these pitfalls, non-invasive assessment for liver fibrosis improvement is more preferred. This can be accomplished by either serological or physical tests^1,38^. Despite the advantages of wide availability, high applicability and good reproducibility, none of the available serological predictive models are liver specific or independent of confounding factors^38^. Hence, serological tests are not commonly used in bariatric surgical literature^39–41^. Physical tests of liver fibrosis consist of ultrasound-based elastography and magnetic resonance-based elastography^38^. Although MRE is highly accurate with low failure rate, it is not justified as a monitoring tool for the concerns of high cost and low availability^17^. Thus, a more readily-available point-of-care tool with lower cost, like TE, should better suit the purpose of follow-up monitoring. TE is a simple and fast examination with immediate result availability, good reproducibility, and low intra-observer and inter-observer variability^38^. Our study demonstrated that TE could be adopted as a longitudinal monitoring tool for the reversal of liver fibrosis after bariatric surgery. Despite a slightly lower compliance rate at 5 years due to lost-of-follow-up, TE had high acceptance among our patients with over 90% compliance rates in the first 4 years.

To the best of our knowledge, our study was the first study to evaluate the long-term treatment responses of NAFLD to bariatric surgery using TE as a monitoring tool. In the literature, TE had been tested on the roles of monitoring disease progression and response to treatment of NAFLD^21–25^. Suzuki et al. reported the changes of LSM over 4 years in 36 patients without paired liver biopsies for monitoring disease progression of NAFLD^21^. Fibrosis progression as reflected by LSM was observed in 25% of patients over 4 years. The changes in LSM were shown to have correlation with the changes in non-invasive fibrosis markers. However, histopathological correlation with paired liver biopsies was not available. Nogami et al. evaluated the disease progression of NAFLD in terms of fibrosis stage using LSM in 34 patients over 10 years^22^. Based on LSM, 32.4% of patients had progression in fibrosis stage and 17.6% had improvement. In 14 out of 34 patients, paired liver biopsies were available and the changes in LSM were shown to have correlation with the change in histological fibrosis stage. In a large scale study of 611 diabetic patients conducted by Lee et al., monitoring by TE could identify fibrosis progression (from LSM < 10 kPa to LSM ≥ 10 kPa) and steatosis progression (from CAP < 248 dB/m to CAP ≥ 248 dB/m) in 4.3% and 52.0% of patients, respectively, over 3 years^23^. As for the longitudinal monitoring of response to treatment for NAFLD, TE had been adopted as a monitoring tool in a few prospective studies^24,25^. Handzlik et al. evaluated the role of metformin in treating 42 patients with NAFLD using CAP and LSM without liver biopsy^24^. Significant reductions in CAP and LSM as surrogate markers for hepatic steatosis and fibrosis were observed in patients receiving metformin over 5 months. Similarly, Shimizu et al. investigated the role of dapagliflozin in treating 57 diabetic patients with NAFLD^25^. Significant improvement in CAP and LSM was observed in patients receiving dapagliflozin over 24 weeks. For the longitudinal response to bariatric surgery, TE had only been evaluated in one short-term follow-up study of 42 patients which showed a significant reduction in median LSM from 8.6 kPa preoperatively to 6.0 kPa at 1 year^42^. Our study similarly demonstrated a significant reduction in LSM at 1 year. Further to this, our study showed that TE was useful in defining sustainable LSM improvement till 5 years after bariatric surgery.

Looking at the factors associated with LSM improvement at 1 year, the effect of bariatric surgery in treating NAFLD was not attributed to weight loss or changes in other body composition parameters. Instead, LSM improvement was only associated with glycemic improvement after bariatric surgery. Both insulin resistance and hyperinsulinemia play pivotal roles in the pathophysiology of NAFLD. Insulin resistance increases adipocyte lipolysis to promote hepatic uptake of circulating free fatty acids. It also augments hepatic de novo lipogenesis by reducing hepatic glycogen storage, increasing gluconeogenesis, and inducing hyperinsulinemia. All these can result in hepatic lipid accumulation and lipotoxicity that further impair insulin signaling, induce oxidative damage, promote inflammation and cause fibrosis within the liver^43^. Bariatric surgery is effective in improving insulin resistance, altering lipid metabolism and reversing inflammatory pathways related to NAFLD^44^. Hence, better improvement in glycemic control after bariatric surgery was associated with LSM improvement. Achieving better postoperative glycemic control was thus the key to treat NAFLD by bariatric surgery.

Earlier studies suggested that the failure rate of TE was lower in obese patients with the use of XL probe^45^. A study by Wong et al. suggested that the median LSM obtained by M and XL probes were nearly identical at each fibrosis stage when M probe was used in patients with BMI < 30 kg/m^2^ and XL probe was used in those with BMI ≥ 30 kg/m^2^^46^. Hence, probe selection during postoperative TE in our study was based on BMI results. While the latest model of TE provides an automated M or XL probe selection based on skin-to-liver capsule distance, our experience suggested that M probe could be readily adopted in providing reliable results in at least 84–100% of patients after bariatric surgery.

We recognize several limitations in this study. First, our patient sample of 37 patients was not large enough to allow direct comparison among different bariatric procedures for the rates of resolution of liver fibrosis. With the additional concerns of loss-to-follow-up from a small sample size, our study also failed to identify any significant association between potential clinical or biochemical variables and long-term LSM improvement at 5 years. Second, this study was conducted with earlier models of TE that did not provide CAP measurement to estimate liver steatosis. Hence, this study was not able to evaluate the rates of resolution of liver steatosis after bariatric surgery. In addition, repeated liver biopsy was not conducted during postoperative follow-up. It was therefore impossible to assess the correlation between LSM improvement and liver biopsy findings. Lastly, our study did not measure the plasma insulin and C-peptide levels for non-diabetic patients. Thus, evaluation of the postoperative changes in insulin resistance (e.g. homeostasis model assessment of insulin resistance) was not possible.

In conclusion, LSM by TE was a useful monitoring tool in assessing the improvement of NAFLD following bariatric surgery. Using LSM for follow-up assessment, bariatric surgery was effective in improving liver fibrosis in a sustainable manner during long-term follow-up.
